# Performance evaluation of commercial short-oligonucleotide microarrays and the impact of noise in making cross-platform correlations

**DOI:** 10.1186/1471-2164-5-61

**Published:** 2004-09-02

**Authors:** Richard Shippy, Timothy J Sendera, Randall Lockner, Chockalingam Palaniappan, Tamma Kaysser-Kranich, George Watts, John Alsobrook

**Affiliations:** 1 GE Heathcare (formerly Amersham Biosciences) Chandler, Arizona 85248, USA; 2 Microarray Shared Service, Arizona Cancer Center, Tucson, Arizona 85724, USA; 3 Child Study Center, Yale University School of Medicine, New Haven, Connecticut 06510, USA

## Abstract

**Background:**

Despite the widespread use of microarrays, much ambiguity regarding data analysis, interpretation and correlation of the different technologies exists. There is a considerable amount of interest in correlating results obtained between different microarray platforms. To date, only a few cross-platform evaluations have been published and unfortunately, no guidelines have been established on the best methods of making such correlations. To address this issue we conducted a thorough evaluation of two commercial microarray platforms to determine an appropriate methodology for making cross-platform correlations.

**Results:**

In this study, expression measurements for 10,763 genes uniquely represented on Affymetrix U133A/B GeneChips^® ^and Amersham CodeLink™ UniSet Human 20 K microarrays were compared. For each microarray platform, five technical replicates, derived from the same total RNA samples, were labeled, hybridized, and quantified according to each manufacturers' standard protocols. The correlation coefficient (r) of differential expression ratios for the entire set of 10,763 overlapping genes was 0.62 between platforms. However, the correlation improved significantly (r = 0.79) when genes within noise were excluded. In addition to levels of inter-platform correlation, we evaluated precision, statistical-significance profiles, power, and noise levels for each microarray platform. Accuracy of differential expression was measured against real-time PCR for 25 genes and both platforms correlated well with r values of 0.92 and 0.79 for CodeLink and GeneChip, respectively.

**Conclusions:**

As a result of this study, we recommend using only genes called 'present' in cross-platform correlations. However, as in this study, a large number of genes may be lost from the correlation due to differing levels of noise between platforms. This is an important consideration given the apparent difference in sensitivity of the two platforms. Data from microarray analysis need to be interpreted cautiously and therefore, we provide guidelines for making cross-platform correlations. In all, this study represents the most comprehensive and specifically designed comparison of short-oligonucleotide microarray platforms to date using the largest set of overlapping genes.

## Background

There are several commercial microarray systems currently available on the market for genome-scale gene expression analysis. Different microarray manufacturers provide distinct underlying technologies, protocols and reagents specific to each system [1]. Despite the widespread use of microarrays, much ambiguity regarding data analysis, interpretation and correlation of the different technologies exists. There is a need for standardization that will facilitate comparison of microarray data from different platforms [2]. Comparison and cross-validation between microarray platforms would greatly increase the understanding and value of the wealth of data generated from each microarray experiment [3]. A number of cross platform comparisons have reported a failure to demonstrate an acceptable level of correlation between different microarray technologies [4-7]. Some of the difficulties in correlating data can be attributed to fundamental differences between cDNA and oligonucleotide based microarray technologies. For example, target preparation differences and single vs. dual labeling techniques complicate the comparisons. Furthermore, cDNA arrays have difficulty in distinguishing between splice variants and highly homologous genes, while oligonucleotide arrays can make these distinctions if designed appropriately. However, when considering oligonucleotide platforms, which have widespread popularity, direct comparisons between different platforms should be less complex and more direct. We assert that differences in platform sensitivity, reproducibility and annotation cross-referencing accuracy account for a majority of the irreconcilable differences previously reported between different platforms [4-7]. When considering these factors we demonstrate a strong correlation between expression ratio data from two different commercially available short oligonucleotide based microarray technologies. This paper provides a comprehensive guideline for microarray analysis, interpretation and cross-platform correlation.

There are two commercially available high-density microarray platforms that use short oligonucleotides for expression profiling. CodeLink (GE Healthcare formerly Amersham Biosciences, Chandler, AZ) and GeneChip (Affymetrix, Santa Clara, CA) microarray platforms utilize oligonucleotide gene target probes of 30 and 25 bases, respectively. Some of the notable differences between the GeneChip and CodeLink systems are, respectively, multiple probes vs. one pre-validated probe per gene target, two-dimensional surface vs. three-dimensional array matrix, and *in situ *synthesized oligonucleotides vs. pre-synthesized, non-contact oligonucleotide deposition. We restricted our comparative analysis to these two platforms because these systems are most similar with respect to oligonucleotide length, target preparation, and single color indirect labeling methodology. Since these commercial assays are similar, and systematic variables were isolated by using the same total RNA starting material for all target preparations, we expected disparity in performance to reflect differences inherent to the microarray platforms. To provide data for comparison of the platforms, five technical replicates of brain and pancreas were processed on each platform and the results were compared for reproducibility, sensitivity, and similarity of results. Standard manufacturer-recommended protocols and settings were employed to obtain the raw data from each platform. In the case of Affymetrix GeneChip, a recent cross-platform microarray evaluation [7] used two additional algorithms [8,9] for analysis of the GeneChip data and found the same level of discordance across the three analysis algorithms as was observed in the cross-platform microarray comparisons [7]. We therefore restricted our analysis of the GeneChip data to the Affymetrix recommended MAS 5.0 software [10]. This methodology was followed to simulate the results users would achieve by following current protocols supplied with each microarray system.

## Results

Two different tissue types were compared in this study to ensure a large number of differentially expressed genes, and provide expression ratios across a wide dynamic range for derivation of the correlation coefficient between the two platforms. The array-to-array precision of each microarray platform was calculated from the five replicates within each tissue sample.

**Figure 1:**
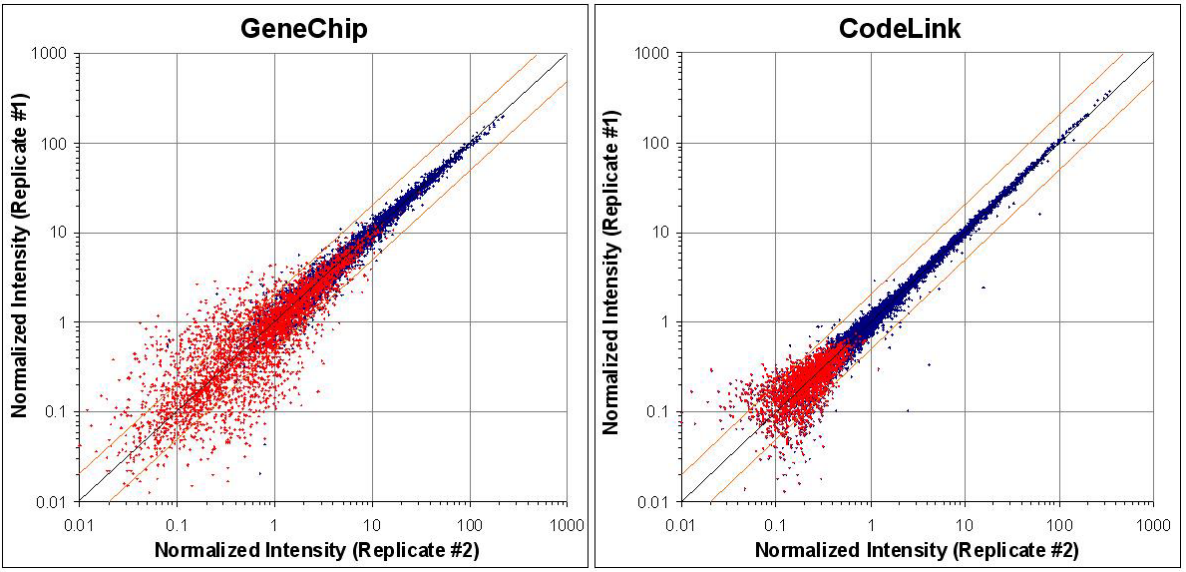
Pair-wise array precision of CodeLink and GeneChip with illustration of respective noise levels. The representative scatter plots show precision of normalized expression values relative to noise. All 10,763 overlapping gene probes are represented in these plots. Values highlighted in red were concordantly 'absent' (noise) calls on both arrays compared. Orange lines show two-fold limits, while the black line represents equality.

The pair-wise array-to-array precision of each microarray platform is illustrated in Figure 1 with respective noise levels for both CodeLink and GeneChip. In these graphs all 10,763 uniquely represented genes, common between both microarray platforms, are illustrated. The GeneChip comparisons display a wider distribution relative to CodeLink at the lower end of the fluorescence detection range. While this wider distribution could be interpreted as indicating a lower level of precision relative to CodeLink, precision should only be assessed for the population of genes with expression values above the noise calculation (i.e. 'present' on the arrays being considered). Due to the variation in noise and specificity level between expression detection systems, each system must individually define its own threshold level cutoff for resultant confidence in signals above technical noise. In addition, in a multi-oligonucleotide detection system, a defined algorithm must be set to determine the method for combining individual probe data to yield a final gene expression level. Therefore, we used each manufacturer's indications for gene signals that should be considered confidently above system noise. The wider distribution observed in the GeneChip platform is within the noise population and therefore should not penalize the overall precision measurements. Qualitatively, CodeLink and GeneChip showed similar levels of precision when concordantly 'absent' genes were excluded within each platform, as illustrated by the blue data points representing the true signal range (Figure 1).

Precision measurements were calculated from signals above noise across the arrays being compared (Tables 1 and 2) to obtain a quantitative assessment. For the five array replicates, within each tissue, a total of 10 pair-wise combinations were made for all genes above noise (i.e. 'present'). Ratios were made in cases where the gene was called 'present' on both arrays being compared. False-change rates of CodeLink and GeneChip were calculated from each pair-wise array comparison between arrays processed with the same starting material. The percentage of ratios derived from the population of concordantly 'present' genes, which fall outside 2-fold (i.e. |log_2 _ratio| > 1), is defined as the false-change rate. Table 1 shows the average and standard deviation of the false-change rate that was calculated for each of the 10 pair-wise array combinations within a sample. The false-change rates between microarray platforms were very similar, however the performance of CodeLink was slightly better with only 0.32% and 0.20% of ratios falling outside 2-fold for brain and pancreas, respectively. GeneChip showed 0.69% and 1.28% ratios outside 2-fold for brain and pancreas, respectively. To assess the level of tightness in the intensity distribution for each platform, we calculated the pair-wise ratio range within which 95% of all ratios fall for each platform (Table 2). For CodeLink, 95% of ratios are below 1.36 and 1.27 for brain and pancreas, respectively. On the other hand, 95% of GeneChip ratios are below 1.49 and 1.64 for brain and pancreas, respectively. Taken together, this data illustrates the precision for CodeLink is slightly higher than GeneChip for both samples tested.

**Table 1:** False-change rate for GeneChip and CodeLink microarray platforms. The false-change rate is defined as the percentage of ratios, derived from the population of concordantly 'present' genes, which fall outside 2-fold (i.e. |log_2 _ratio| > 1). The table above contains the average and standard deviation of the false-change rate, calculated across the 10 pair-wise array combinations within a sample. False-change rate was calculated from signals above noise across the arrays being compared.

**Array Platform**	**Tissue**	**AVG**	**STDEV**
CodeLink	Brain	0.32%	0.13%
	Pancreas	0.20%	0.14%
GeneChip	Brain	0.69%	0.27%
	Pancreas	1.28%	0.17%

**Table 2:** Precision ratio summary for GeneChip and CodeLink microarray platforms. Precision measurements were calculated from signals above noise across the arrays being compared. For CodeLink, there were 7,882 and 6,603 ratios, on average, for each pair-wise array-to-array comparison, within brain and pancreas respectively. For GeneChip, there were 6,734 and 5,137 ratios, on average, for each pair-wise array-to-array comparison, within brain and pancreas respectively. For each of the 10 pair-wise combinations, the ratio range within 95% of the ratios fall was calculated. This table contains the average and standard deviation, in which 95% of ratios fall within, across all 10 pair-wise array combinations within a sample.

**Array Platform**	**Tissue**	**AVG**	**STDEV**
CodeLink	Brain	1.36	0.09
	Pancreas	1.27	0.01
GeneChip	Brain	1.49	0.05
	Pancreas	1.64	0.03

**Figure 2:**
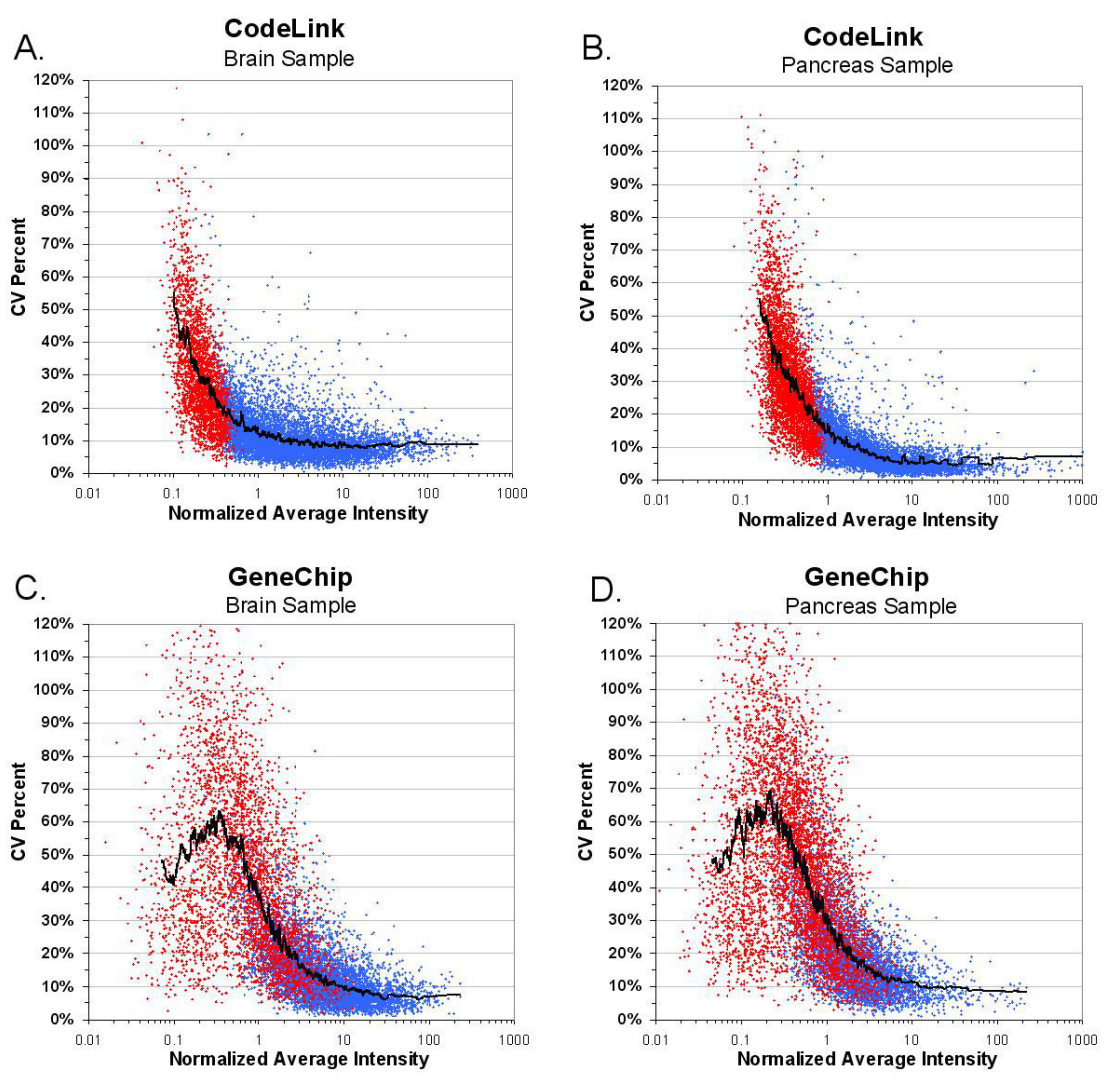
Coefficients of variation for each platform as a function of intensity, across all replicates. Genes which are concordantly 'absent' are shown in red. The black line represents the 100-probe moving average.

In addition to pair-wise array precision, we calculated coefficients of variation (CV) for each platform as a function of intensity, across all replicates. In Figure 2, CV is represented as a percentage calculated as the gene's signal standard deviation divided by mean signal across all array replicates. Genes that are concordantly 'absent' are shown in red. Concordantly 'absent' refers to genes called 'absent' by the manufacturer's software on all 5 replicate arrays tested. The black line represents the 100-probe moving average of all data points. The precision of all 'present' signals is similar between CodeLink and GeneChip, as illustrated by the moving-average level within the blue region. The median percent CV for the population of 'present' genes was 8% for both platforms. However, as gene intensity decreases, the average variance increases earlier in the distribution for GeneChip relative to CodeLink, as illustrated by the 100-probe moving average, at the boundary between red and blue data points. It is expected that variance would naturally increase at this boundary and since the rise in variance coincides with the level of concordantly 'absent' signals, demonstrating that noise is more than likely being identified correctly by each platform's image quantification software. Notably, Figures 1 and 2 illustrate a higher level of noise for GeneChip relative to CodeLink.

**Figure 3:**
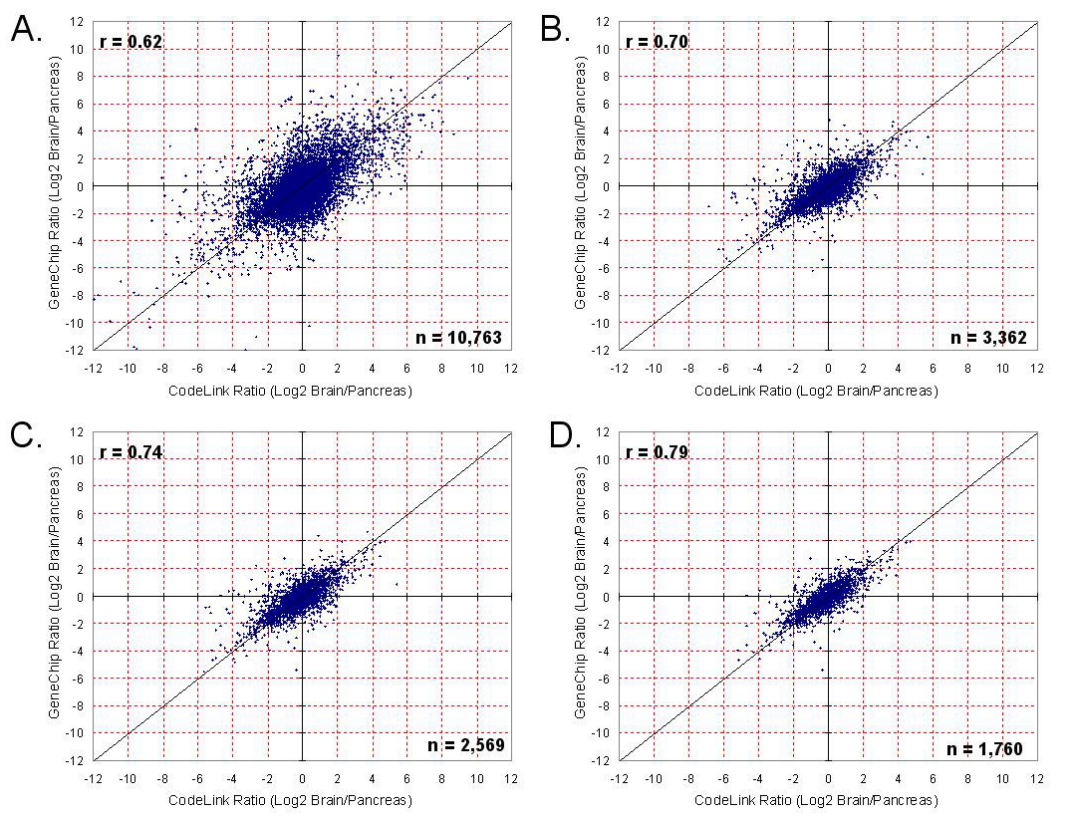
Correlation of differential expression ratios between CodeLink and GeneChip. Pearson correlation coefficients (r) are shown for each comparison. (A.) When all 10,763 overlapping genes are compared between platforms the correlation is 0.62. (B.) All values for genes concordantly 'absent' were removed prior to making the cross-platform correlation. In this case, 3,362 genes are called 'present' on at least 1 of the 5 replicates across both tissues and platforms. (C.) 2,569 genes called 'present' on at least 3 of the 5 replicates across both tissues and platforms. The correlation improves further to 0.74. (D.) Genes called present on all 5 replicates across both tissues and platforms. For these 1,760 genes the correlation is 0.79.

Differential expression ratios were compared between platforms to determine the cross-platform correlation. As shown in Figure 3, when all 10,763 uniquely overlapping genes are compared between platforms, the correlation is weak (r = 0.62, where 'r' represents the Pearson correlation coefficient). However, when removing the population of concordantly 'absent' signals, the correlation is r = 0.70 between microarray platforms. When limiting the comparison to those values which are called 'present' on at least 3 of the 5 replicates across tissues and platforms, the correlation improves further to 0.74. If we further limit our comparison to only genes called concordantly 'present' (i.e. 'present' on all 5 replicates across both tissues and platforms) the correlation r = 0.79.

**Figure 4:**
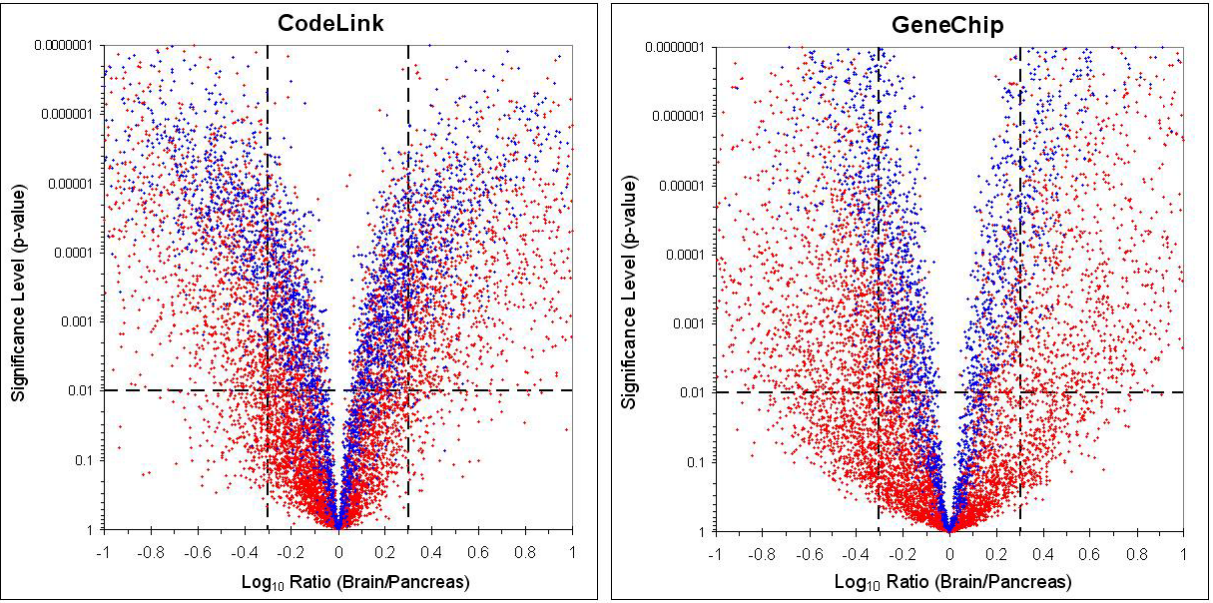
'Volcano plots' for CodeLink and GeneChip. Each point represents a gene from the uniquely common set of 10,763 genes between platforms. Data points highlighted in blue represent genes which are concordantly 'present' in both tissues. The log_10 _ratio of expression (brain/pancreas) is shown on the x-axis and the p-value, from a two-tailed Student's t-tests on normalized log-transformed intensities, is shown on the y-axis. The vertical dashed lines represent 2-fold change ratios and the horizontal dashed line represents the statistical-significance level where p = 0.01.

The improvement in the correlation coefficient from 0.62 to 0.79 achieved by excluding noise underscores the value in identifying the population of signals above noise for cross-platform comparisons. The 'volcano plots' in Figure 4 further confirm this point. Each data point represents a probe from the uniquely common set of 10,763 gene probes between the platforms relative to ratio and significance value. Data points highlighted in blue represent genes that are concordantly 'present' in both tissues. Hence, these blue data points are the genes called 'present' on all replicates across both tissues (n = 10). The mean log_10 _ratio of expression (brain/pancreas) is shown on the x-axis and the p-value, from a two-tailed Student's t-tests on normalized log-transformed intensities, is shown on the y-axis. The vertical dashed lines represent 2-fold change ratios, which are commonly used in the field as significance levels for non-replicated array experiments. The horizontal dashed line represents the statistical-significance level where p = 0.01 (an uncorrected lenient level, used to error on the side of inclusion). The lower right- and left-hand corners of each graph contain the genes that showed a large fold-change but fail to achieve statistical significance (p > 0.01). GeneChip results show a larger number of genes in these regions as compared to the CodeLink data. The data points located in the upper-central region of each graph represent genes that were statistically significant (p < 0.01) despite modest fold-changes (< 2-fold). The minimal-detectable statistically significant fold-change was tighter for CodeLink relative to GeneChip as illustrated by the distance across the 'volcano' plot at the 0.01 significance level. In addition, the number of genes above the 0.01 significance level was greater for CodeLink relative to GeneChip. The distribution difference between the red and blue data points demonstrates the advantage of identifying signals above noise for making ratio calculations.

**Figure 5:**
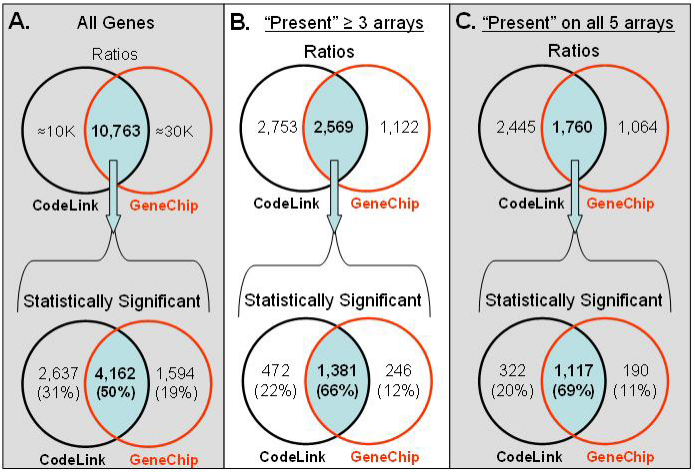
Venn diagrams of differential expression calls and statistical significance across both microarray platforms. A two-sample two-tailed t-test on normalized log-transformed intensities was performed for each microarray platform. (A) The entire set of 10,763 uniquely common genes between platforms was used to determine the number of statistically significant (p < 0.01) expression ratios. Genes above and below noise were included in the analysis. (B) Statistical significance determined from the set of 2,569 genes which are 'present' on at least 3 arrays in both tissues. Expression values below noise ('absent') were not included in the analysis. (C) Statistical significance determined from the set of 1,760 genes which are 'present' on all 5 arrays in both tissues (i.e. concordantly 'present').

The 'volcano' plots are translated into Venn diagrams of statistically significant differentially expressed genes for each platform in Figure 5. Statistically significant (p < 0.01) expression ratios were determined using the entire set of 10,763 uniquely common genes between platforms. The total number of statistically significant differentially expressed genes detected by both platforms from this common set was 8,393. The intersection of the two platforms represents 50% of the total number of significantly differentially expressed genes. It is important to note that using the method described here, only probes considered above system noise are utilized for the correlation calculation. This leaves a set of probes which are discrepant calls and require further analysis to determine the accuracy of detection. The CodeLink platform called 5,322 genes concordantly present across the two tissues while the GeneChip platform called 3,691 genes (figure 5B, top panel). The union represents 2,569 concordantly present calls common to both platforms, where n = 3 or more. In addition, the set of 1,760 concordantly 'present' gene probes, across both platforms and tissues, was used to create a Venn diagram of ratios derived from signals concordantly above noise. The intersection of the two platforms represents 69% of the total number of differentially expressed genes. There are a higher percentage of commonly significantly changed genes between platforms when noise is excluded from ratio calculations. In both cases CodeLink shows a larger percentage of statistically differentially expressed genes at a p value less than 0.01.

**Figure 6:**
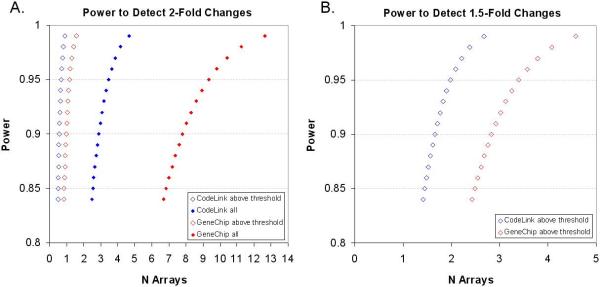
Power analysis estimating the number of technical array replicates needed to achieve a reasonable level of statistical power or confidence for CodeLink (blue) and GeneChip (red) when noise was included (solid diamonds) or excluded (open diamonds). For both graphs the alpha was set at 0.01. (A) Relationship between power and arrays necessary to statistically discriminate two-fold changes in expression. To achieve a power of 0.90 using all 10,763 genes, 3 arrays are minimally necessary for CodeLink while 8 are required for GeneChip. However, when noise is excluded, both GeneChip and CodeLink require only 1 array to achieve this same level of power. In fact, when noise is excluded, 1 array for both GeneChip and CodeLink has a power of 0.99 to detect two-fold changes in expression. B.) In order to detect 1.5 fold changes in expression, at a 0.90 power, when noise is excluded, CodeLink minimally requires 2 arrays while GeneChip requires 3.

A power analysis was conducted on each microarray platform to estimate the number of technical replicates needed to achieve a reasonable level of statistical confidence when noise was either included or excluded from the dataset (Figure 6). Evaluating the power of each platform at the level of technical replication allows researchers to gauge the underlying system variance before introducing biological variance in their studies. From our analysis, to achieve a power of 0.90 using all 10,763 genes, 3 array replicates are minimally necessary for CodeLink while 8 replicates are required for GeneChip. However, when noise is excluded, both CodeLink and GeneChip require only 1 array to achieve this same level of power. In fact, when noise is excluded, 1 array for both GeneChip and CodeLink has a 0.99 level of power in detecting two-fold differences in expression. The significant improvement in power by excluding noise provides considerable value to microarray users since fewer arrays are required to resolve desired differences in expression. By identifying and removing noise both systems can detect differential expression ratios less than 2-fold with a high level of power. However, more genes are lost on the GeneChip platform as a result of the higher level of noise relative to CodeLink. Additionally, when noise is excluded, 1.5-fold changes in expression can be detected, at a 0.90 power, using 2 CodeLink or 3 GeneChip technical replicates.

**Figure 7:**
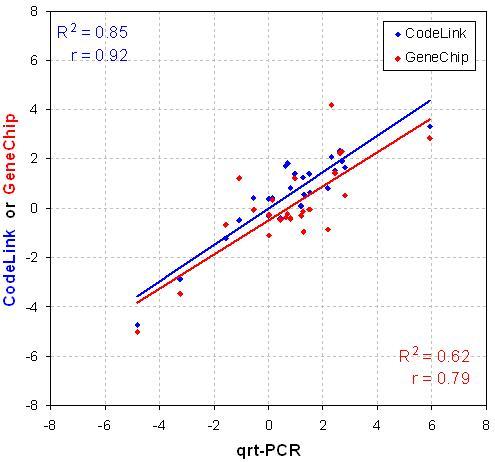
Accuracy of CodeLink and GeneChip differential-expression ratios relative to qrtPCR. Expression ratios for each microarray platform were measured against results from qrtPCR for a randomly selected subset of 25 genes. Pearson correlation coefficients (r) are shown for each comparison.

The accuracy of CodeLink and GeneChip differential-expression ratios were compared to quantitative real-time PCR (qrtPCR). Microarray expression ratios were measured against results from qrtPCR for a randomly selected subset of 25 genes (Table 3) and plotted in Figure 7. Both microarray platforms correlated well to this alternative expression-profiling technology with Pearson correlation coefficients of 0.92 and 0.79 for CodeLink and GeneChip, respectively.

## Discussion

Increased access and utilization of microarray data through core facilities and affordable commercial microarray systems is driving the need for direct comparisons of data between the different available platform technologies. The ability to exchange data across different platforms gives the research community the ability to cross-validate results and extend understanding of biological processes through integration of published data collected with different technologies. The results presented here demonstrate that we are closer to reaching this goal than previously reported [4-7].

We have compared two commercial platforms and in doing so present several steps required for making comparisons between short oligonucleotide microarray data sets. First, one must normalize annotation. Unfortunately, despite the completion of the human, rat and mouse genome sequencing projects, accurate and stable gene annotation information is not available. The existence of inaccurate sequence information, absence of an exact gene count, incomplete understanding of splicing variations, and the complexity of highly homologous gene sequences all contribute to the challenges of generating a controlled vocabulary for uniquely and constantly annotating genes at the present time. In addition, when considering commercially available arrays, the consumer is left to rely on the manufacturer to provide a probe with a one to one correlation to the intended gene target. Furthermore, until recently manufacturers have withheld the release of the exact probe sequences to researchers [7]. Now that with a simple disclosure agreement probe sequences from the major manufacturers are readily available to the users, discrepancies in some results will be explained by differences in actual probe and probe sets targets as defined by sequence homology. Some probes target different or multiple splice variants and some probes are not specific to a single gene, but instead, target multiple homologous genes. Since the use of GeneChip probe sequences for deriving inter-platform overlap is currently prohibited by Affymetrix for publication purposes, we needed to rely upon public annotation to determine the overlap between products rather than more informative sequence-based comparisons. We believe that the use of probe sequences will help to further refine the accuracy of the gene overlap set, and increase the already strong correlation between platforms demonstrated here. In addition, without the use of sequence information, we filtered the data to include only those probes and probe sets that identify a specific gene target or common regions of splice variants of a single gene target. Both manufacturers in some cases carry multiple probes or probe sets per target gene. Trying to determine which probes to compare in this case without the use of sequence information is nearly impossible. Therefore, only uniquely represented gene probes by both manufacturers were used for comparisons. By employing this conservative methodology, we reduce the risk of inappropriately comparing data from probes designed to detect different transcripts or genes despite having a similar annotation. Importantly, we used a common build of UniGene cluster IDs to find unique gene probes which overlap between the two products.

When comparing between the two platforms using tissue ratio data without regard for noise, the correlation between platforms is not very strong (r = 0.62, Figure 3A), similar to what was reported by Tan *et al. *2003 [7]. This brings us to the second step, removing background signals. Considering background noise has random sources and sources that are different in nature for the two platforms, one would not expect to find a strong correlation when using noise values in platform comparisons. Each manufacturer warns users to be critical of confidence in calls that are below the defined threshold or considered 'absent'. Therefore, we removed noise and made correlations based only on calls that were 'present' in both tissue samples and microarray systems. Kuo *et al. *made a limited but similar attempt to reduce noise by using what they termed a "variance filter" [4]. Our process of filtering noise reduced the overlap of 10,763 genes to 3,362, 2,569 or 1,760 genes if one accepts 'present' calls on at least 1, 3 or all 5 of the array replicates, respectively, across both tissues and platforms. Using this methodology, however, we found a stronger ratio correlation between the two platforms (r = 0.70, 0.74 or 0.79, Figure 3B,3C,3D). We have found that when limiting the comparison set to those probes which are uniquely represented, specific for their targets of interest, and called 'present' in the samples tested on each platform, the correlation between technologies is very reasonable for data sharing. Supporting this methodology, a recent study found a substantial improvement in the correlation between spotted long-oligo arrays and the Affymetrix platform with data filtering by removing low intensity signals below the median [11]. Interestingly, when Barczak and colleagues removed low intensity signals, the Pearson correlation coefficient improved from 0.60 to 0.80, which is in the same range as in our study [11]. Rather than removing all low intensity signals below the median, we recommend data filtering by using each manufacturer's standard software package to identify those genes which are within noise. This approach to filtering noise offers great value to microarray users since our recommendation does not require the immediate loss of 50% of the data in making cross-platform comparisons.

Finally, an alternative expression-profiling technology, qrtPCR, was used to follow up on a smaller subset of the concordantly correlated set to demonstrate that the data generated here was not merely an anomaly specific to oligonucleotide arrays (Figure 7). Both platforms correlated well to this alternative expression-profiling technology with r values of 0.92 and 0.79 for CodeLink and GeneChip, respectively. Previous studies have found agreement between genes screened with microarray technology and subsequent qrtPCR verification of those expression measurements [12,13]. We are in the experimental process of using qrtPCR with a larger set of genes as an independent method to resolve discordant gene expression results between the two microarray platforms.

The comparison described here parses the data into three sets: *(1.) *Concordantly 'present' which was used to calculate the correlation comparisons; *(2.) *Concordantly 'absent', where both platforms agree that the transcript is not 'present' in the samples tested; and *(3.) *'Present' on one microarray platform but not the other, which are considered a separate set of discrepant results. In the studies presented here, the CodeLink platform generates a higher percentage of detectable signals above noise (Figures 1, 2, and 5). This finding is consistent for all replicate arrays across both tissues analyzed. Previously, Ramakrishnan *et al. *2002 reported detection down to an estimated sensitivity level between 1:750,000 and 1:900,000 for the CodeLink platform [14]. However, biological validity of these low level calls by qrtPCR or other method have not been confirmed the results. In addition, a significant number of signals were detected by the GeneChip platform and were not detected by the CodeLink platform. Therefore, follow up studies are necessary to definitively determine which of the discordant calls are biologically relevant and which may be potential false positive calls. It would be informative to understand the underlining basis of the discordant calls. Assigning cause such as differences in sensitivity, analysis algorithms, or characteristics of the two platforms would be of great values to furthering comparative studies.

Discrepant calls between the two platforms may derive from differences in the GeneChip and CodeLink platform technologies. The platforms differ in the oligodeoxyribonucleotide probe length and number of probes per gene. A microarray study, using covalently attached oligodeoxyribonucleotides, found that 30- and 35-mer oligodeoxyribonucleotides generated signals two- to five-fold higher than 25-mers [15]. Relogio *et al. *suggested that 30-mers offered the best compromise between sensitivity and specificity [15]. However, the GeneChip platform offers multiple probes per gene, potentially offsetting the need for longer probes through multiple hybridization points. The CodeLink platform contains one pre-validated probe per gene that was screened for performance from an original panel of three probes per gene. Previous research has demonstrated that one probe per gene is sufficient to accurately measure differential expression [16]. Having one pre-validated probe per gene rather than a panel of probes per gene on a microarray platform may be advantageous towards improving sensitivity since there is no requirement that many probes within a gene must agree for expression to be detected and called. A single probe must, however, be very accurately designed to cover the range of splice variants feasible, and must reside in an area accessible to the RNA or DNA fragments hybridizing. Variation in signals may also derive from the nature of the substrate for probe attachment. Previous publications have indicated that the use of a three-dimensional matrix coated slide results in a larger number of potential attachment sites than modified glass [17-19]. Stillman and Tonkinson [20] have shown higher specific hybridization signals on a three-dimensional matrix compared with glass. In addition, it has been demonstrated that the CodeLink three-dimensional matrix allows for reduced steric hindrance and increased availability of the entire oligonucleotide for hybridization with its intended target [21]. Side-by-side comparisons of the performance of the same probe set and analysis technique would be required to confirm any contribution to discrepant results observed in this study.

Discrepant calls between the two platforms may also likely derive from differences in the GeneChip and CodeLink analysis algorithms. The use of mismatches on the GeneChip platform may limit detection since others have reported that, in general, one third of GeneChip mismatches are higher in signal than their perfect match counterparts [9,22,23]. Alternative analysis methodologies that do not utilize the mismatch controls may alter the discordant set, but as described earlier, there is a large potential variation in the different methodologies and a lack of a single majority method. Therefore, we chose to analyze the dataset in this study with the MAS 5.0 algorithms, as recommended by Affymetrix. It is likely that each of the aforementioned factors, in addition to annotation differences, contribute to variable results, and taken together account for the set of discrepant calls observed between the GeneChip and CodeLink platforms (Figure 5).

## Conclusions

This paper highlights the value of separating signal from noise in order to improve microarray cross-platform correlations. We also demonstrate a stronger correlation between platforms than previously reported based on our data filtering and parsing methodology. We believe there is strong similarity in calls by each system and differences in sensitivity and levels of noise are largely responsible for lower levels of correlation. Furthermore, as a standardized annotation system develops and freely open access to the use of microarray probe sequences is realized, it will help clear up discrepancies on a case by case basis.

## Methods

### Array design and fabrication

CodeLink UniSet Human 20 K Bioarray (Amersham Biosciences, Chandler, AZ) contains a collection of approximately 20,289 probes within a single reaction chamber on each individual slide. All oligonucleotide probes are 30 bases in length. The core of the CodeLink platform is a glass slide coated with a polyacrylamide gel matrix to create a three-dimensional aqueous hybridization environment. Modified 5'-amine-terminated oligonucleotides are deposited onto the polymer using piezoelectric dispensing robots and then covalently attached to activated functional groups within the gel matrix. Oligonucleotides are co-dispensed with a fluorescein-derivative dye, which enables scanning and inspection of every feature element on every slide after the dispensing. Additional sites are then blocked and slides are washed, rinsed and dried prior to attachment of an integrated, proprietary, polypropylene hybridization chamber. All probes appearing on the final product have been pre-validated for performance and screened from an original panel of up to three probes per gene.

The HG-U133 GeneChip Set from Affymetrix (Santa Clara, CA, USA) contains 44,928 probes, on 2 chips, that represent 42,676 unique sequences from the GenBank database corresponding to 28,036 unique UniGene clusters. The GeneChip technology is based on a photolithographic *in situ *synthesis. Individual probes consist of 25 base DNA sequences.

### Target preparation and array hybridization

One lot of human brain and pancreas total RNA (brain lot#033P010402009A and pancreas lot#022P0102B from Ambion) was assessed for quality using the Agilent 2100 Bioanalyzer and split equally between Amersham Biosciences in Chandler, Arizona and the Genomics Shared Service at the Arizona Cancer Center. The Affymetrix target preparations and hybridizations were performed entirely at the Arizona Cancer Center to ensure that these microarrays were run by an independent party with GeneChip expertise. In addition, an aliquot from these lots of total RNA was saved and subsequently used in qrtPCR reactions for verifying the expression profiles obtained by each microarray platform.

For each Affymetrix GeneChip, double-stranded cDNA was synthesized from 5 ug of total RNA with the SuperScript Double-Stranded cDNA Synthesis Kit (Invitrogen) and dT24-T7 primer (Operon) according to the manufacturer's instructions. Biotin-labeled cRNA was prepared by *in vitro *transcription using the BioArray High Yield RNA Transcript Labeling Kit (Enzo). The dsDNA was mixed with 1× HY reaction buffer, 1× biotin labeled ribonucleotides (NTPs with Bio-UTP and Bio-CTP), 1× DTT, 1× RNase inhibitor mix and 1× T7 RNA polymerase. The mixture was incubated at 37°C for 5 hours. The labeled cRNA was then purified using an RNeasy mini kit (Qiagen) according to the manufacturer's protocol and ethanol precipitated. Fragmentation of cRNA, hybridization, washing, staining, and scanning were performed as described in the Affymetrix GeneChip Expression Analysis Technical Manual [24]. Briefly, the purified cRNA was fragmented in 1× fragmentation buffer (40 mM Tris-acetate, 100 mM KOAc, 30 mM MgOAc) at 94°C for 35 minutes. For hybridization with GeneChip cartridge (Affymetrix), 15 ug of fragmented cRNA was incubated with 50 pM control oligonucleotide B2, 1× eukaryotic hybridization control (1.5 pM *BioB*, 5 pM *BioC*, 25 pM *BioD*, and 100 pM *cre*), 0.1 mg/ml herring sperm DNA, 0.5 mg/ml acetylated BSA and 1× manufacturer recommended hybridization buffer, and hybridization was performed with a GeneChip Fluidic Station (Affymetrix) using the appropriate antibody amplification, washing and staining protocol. The phycoerythin-stained array was scanned, resulting in a digital image file. In all, 5 replicates of U133A and U133B were processed for each total RNA sample. Therefore, 10 target preparation reactions were performed for each of the two tissues to generate the necessary cRNA for this study.

For each CodeLink Bioarray, double-stranded cDNA and subsequent cRNA was synthesized from 5 ug of total RNA using the CodeLink Expression Assay Kit (Amersham Biosciences) according to manufacturer's instructions [25]. Briefly, cRNA was prepared by *in vitro *transcription using a single, labeled nucleotide, biotin-11-UTP in the IVT reaction at a concentration of 1.25 mM. Unlabeled UTP was present at 3.75 mM, while GTP, ATP, and CTP were at 5 mM. The mixture was incubated at 37°C overnight for 14 hours. The labeled cRNA was then purified using an RNeasy^® ^mini kit (Qiagen). Fragmentation of cRNA, hybridization, washing, staining, and scanning were performed as described [26]. Briefly, the purified cRNA was fragmented in 1× fragmentation buffer (40 mM Tris-acetate pH 7.9, 100 mM KOAc, 31.5 mM MgOAc) at 94°C for 20 minutes. For hybridization with CodeLink bioarrays (Amersham Biosciences), 10 ug of fragmented cRNA in 260 ul of hybridization solution was added to each bioarray via the Flex Chamber port and incubated for 18 hours at 37°C, while shaking at 300 r.p.m. in a New Brunswick Innova™ 4080 shaking incubator. The 10 bioarrays, in this study, were processed in parallel using the CodeLink Shaker Kit and CodeLink Parallel Processing Kit (Amersham Biosciences). Bioarrays were stained with Cy5™-streptavadin (Amersham Biosciences) and scanned using a GenePix^® ^4000 B scanner (Axon Instruments).

### Deriving expression values and classifying probes within noise ('absent') for each platform

For the U133 GeneChip technology, each gene is represented by 11 probe pairs containing both a perfect match probe (PM) and a mismatch probe (MM) where the middle (13^th^) base of each 25-mer probe is incorrect. The MM probe is designed to give an indication of the degree of nonspecific hybridization [26]. The MAS 5.0 software uses both PM and MM values for the expression calculation, one that avoids the production of negative values. MAS 5.0 employs a scenario-based approach to expression calculations and in general hypothesizes that MM probes should show lower hybridization signal than the corresponding PM probes. A decision process is used when this PM > MM assumption is broken. When all MM values are less than their PM counterparts, an expression value is calculated using a one-step bi-weight estimate of the log(PM – MM) values for each probe pair. However, when the MM value for a probe pair is greater than the PM value, two differing scenarios are applied. 1.) If the values of the PM probes are sufficiently large and separable from the background and MM signals, then the MM value is replaced with a value calculated as typical for the probe set. 2.) If it is difficult to separate the probe signals from background then the MM signal is substituted with a value slightly less than the PM signal. Once an expression value is calculated for each probe set the next step is the calculation of a Detection *p*-value and the comparison of each Discrimination score to the user-definable threshold (Tau). Tau is a small positive number that can be adjusted to increase or decrease sensitivity and/or specificity of the analysis (default value = 0.015). The One-sided Wilcoxon's Signed Rank test is the statistical method employed to generate the Detection *p*-value. It assigns each probe pair a rank based on how far the probe pair discrimination score is from Tau. The user-modifiable Detection *p*-value cut-offs, Alpha 1 (α1) and Alpha 2 (α2) provide boundaries for defining 'Present', 'Marginal' or 'Absent' calls. At the default settings (α1 = 0.04 and α2 = 0.06), any p-value that falls below α1 is assigned a 'Present' call, and above α2 is assigned an 'Absent' call. 'Marginal' calls are given to probe sets which have *p*-values between α1 and α2. In our study, the MAS 5.0 default parameters were retained. For a complete description of the MAS 5.0 algorithms and statistical tests please refer to the Affymetrix manuals [10,27,28].

For the CodeLink bioarrays, spot signals are quantified using ImaGene 5.5 software (BioDiscovery, Marina Del Ray, CA). The mean intensity is taken for each spot and background corrected by subtracting the surrounding median local background intensity. A spot is considered 'absent' (within noise) if the spot's signal mean is less than its corresponding local background mean plus one standard deviation of local background pixels. For each probe the local background is comprised of a circular area of pixels surrounding the segmented signal. The image segmentation and quantification process is outlined in the ImaGene 5.5 user's manual [29].

### Cross-platform comparisons of expression data

To facilitate comparisons between data sets, CodeLink probes and GeneChip probe sets were mapped to specific sequence clusters according to the NCBI Human UniGene build #166 relative to the manufacturer's provided NCBI accession numbers. Multiple probe or probe sets targeting a single UniGene cluster or single probe or probe sets targeting multiple clusters were removed from consideration. The overlapping and uniquely represented UniGene clusters were used to identify 10,763 gene probes for comparison between platforms. Gene-expression values were global linearly normalized according to manufacturers' standard normalization procedure [9,26]. The 96% trim-mean of the entire GeneChip array was used for Affymetrix normalization while CodeLink values were normalized against the array median. The globally normalized data from both platforms were scaled to 1.0 in order to bring both platforms to the same intensity range for comparative purposes. The analysis was performed using SAS statistical software and Microsoft Excel.

### Power analysis of CodeLink and GeneChip platforms

A power analysis is a computational tool used to determine the replication needed to achieve a desired level of confidence in results from a particular experiment [30-32]. Determining the number of microarray replicates necessary for classification of expression profiles has been presented as an important issue [33,34] and should be one of the first things to consider when designing any experiment. Fore each tissue we hybridized the same target on each of five microarrays; therefore the expected fluorescence values for each independent probe should be the same from each array to array replicate, making the expected fold change equal to 1 (i.e. μ_1 _= μ_2_). The power analysis was modeled from log_2 _transformed ratios derived from all pair-wise array-to-array combinations across the five replicates within the brain sample, since this tissue had the greatest similarity in performance between microarray platforms. Expression profiling of the pancreas sample showed many more genes within noise ('absent') for the GeneChip platform relative to CodeLink. The power analysis was conducted as previously described [35,36] for the population of all 10,763 genes within each platform and the population of genes above noise ('present').

### Real-time PCR

The TaqMan^® ^One-Step RT-PCR Master Mix Reagent Kit (Applied Biosystems, Foster City, CA, USA) was used with each custom designed, gene-specific primer/probe set to amplify and quantify each transcript of interest. Optimal primer/probe sets were selected using Primer Express software version 1.0 B6 (Applied Biosystems). Reactions (25 ul) contained 100 ng of total RNA, 300 nM forward and reverse primers, 200 nM TaqMan probe, 12.5 uL 2X Master Mix without the enzyme uracil DNA glycosylase (UNG), 0.625 mL MultiScribe™ and RNAase Inhibitor Mix, and 6.875 uL RNAse-free water. RT-PCR amplification and real-time detection were performed using an ABI PRISM 7700 Sequence Detection System (Applied Biosystems) for 30 min at 48°C (reverse transcription), 10 min at 95°C (AmpliTaq Gold activation), 38 cycles of denaturation (15 s at 95°C), and annealing/extension (60 s at 60°C). Data were analyzed using ABI PRISM Sequence Detection Software version 1.6.3 and then further processed using Microsoft^® ^Excel (Microsoft, Redmond, WA). Cyclophilin (PPIE) served as the endogenous control for the normalization of input target RNA. Raw C_T _values, qrtPCR primer/probe sequences, and corresponding array probe names are available in supplementary material [see additional files 1, 2, and 3, respectively].

## Competing interests

RS, TJS, RL, TK-K and CP are employees of GE Healthcare.

## Authors' Contributions

RS planned and designed the study, conducted the micoarray experiments with the CodeLink platform, analyzed the data, generated all of the figures, and drafted the paper. TJS helped with writing of the paper, deriving the overlap between platforms, provided overall technical guidance and coordination. RL designed, conducted, and analyzed the quantitative PCR experiments, performed bioinformatics support, and edited the manuscript. CP read the manuscript and provided comments. TK-K edited the manuscript. GW helped with the experimental design and was responsible for generating all of the GeneChip data as well as editing the manuscript. JA provided guidance with the statistical power analysis and additions to the manuscript. All authors read and approved the final manuscript.

**Table 3:** List of genes evaluated using qrtPCR. For each gene, the microarray and qrtPCR brain/pancreas log_2 _ratios are listed. Raw C_T _values, qrtPCR primer/probe sequences, and corresponding array probe names are available in supplementary material [see additional files 1, 2, and 3, respectively].

**Gene**	**NCBI Acc**	**Description**	**qrtPCR**	**CodeLink**	**GeneChip**
MLP	NM_023009.1	MARCKS-like protein	2.33	2.08	4.20
COX7A2L	NM_004718.1	cytochrome c oxidase subunit VIIa polypeptide 2 like	1.19	0.10	-0.31
COL6A3	NM_004369.1	collagen, type VI, alpha 3, transcript variant 1	-4.80	-4.75	-5.02
PRDX3	NM_006793.1	peroxiredoxin 3, nuclear gene encoding mitochondrial protein	0.82	0.81	-0.42
CDKN1A	NM_000389.1	cyclin-dependent kinase inhibitor 1A	-3.25	-2.89	-3.47
NUTF2	NM_005796.1	nuclear transport factor 2	1.25	1.25	-0.12
CEBPD	NM_005195.1	CCAAT/enhancer binding protein, delta	-0.54	0.41	-0.07
COL9A3	NM_001853.1	collagen, type IX, alpha 3	2.62	2.35	2.22
GLDC	NM_000170.1	glycine dehydrogenase	5.93	3.33	2.85
TGFA	NM_003236.1	transforming growth factor, alpha	2.44	1.54	1.43
GALK2	NM_002044.1	galactokinase 2	0.64	1.73	-0.39
ESR1	NM_000125.1	estrogen receptor 1	0.03	0.39	-0.28
FMO3	NM_006894.2	flavin containing monooxygenase 3	-1.06	-0.49	1.22
AKT1	NM_005163.1	v-akt murine thymoma viral oncogene homolog 1	0.14	0.40	0.34
PRPS1	D00860.1	phosphoribosyl pyrophosphate synthetase subunit I	1.53	0.62	-0.04
RPA3	NM_002947.1	replication protein A3	1.30	0.57	-0.97
SLIT2	NM_004787.1	slit homolog 2 (Drosophila)	2.81	1.64	0.51
HIC	AF054589.1	HIC protein isoform p40 and HIC protein isoform p32	0.70	1.83	-0.22
HSA275986	NM_018403.1	transcription factor SMIF	1.51	1.41	-0.06
TFCP2L1	NM_014553.1	transcription factor CP2-like 1	-1.55	-1.23	-0.66
PPIE	NM_006112.1	peptidylprolyl isomerase E (cyclophilin E)	0.00	-0.29	-1.10
FLJ14800	NM_032840.1	hypothetical protein FLJ14800	0.97	1.41	1.20
MGC24039	AL137364.1	cDNA DKFZp434E0626	2.70	1.92	2.29
USF1	X55666.1	late upstream transcription factor	2.18	0.81	-0.86
B4GALT7	NM_007255.1	xylosylprotein beta 1,4-galactosyltransferase, polypeptide 7	0.45	-0.41	-0.44

## Supplementary Material

Additional File 1 This file contains gene names, sample designations, C_T _values for triplicates, mean C_T _values, median C_T _values, standard deviation of replicates, and coefficient of variation of replicates for each gene evaluated by qrtPCR. Click here for file

Additional File 2 This file contains gene names, oligonucleotide sequences, and oligonucleotide type (FORWARD PRIMER, HYB OLIGO, and REVERSE PRIMER) for each gene evaluated by qrtPCR. Click here for file

Additional File 3 This file contains gene names, descriptions, GenBank accessions, log2 ratios (qrtPCR, CodeLink, and GeneChip), CodeLink probe names, and GeneChip probe set names for each gene examined by all three technologies. Click here for file
